# Case Report: “Aggressive” perioperative antiseizure medication prophylaxis in patients with glioma-related epilepsy at high risk of early postoperative seizures following awake craniotomy

**DOI:** 10.3389/fsurg.2023.1282013

**Published:** 2024-01-11

**Authors:** Brin E. Freund, Kurt Jaeckle, Alfredo Quinones-Hinojosa, Anteneh M. Feyissa

**Affiliations:** ^1^ Department of Neurology, Mayo Clinic Florida, Jacksonville, FL, United States; ^2^ Department of Neurosurgery, Mayo Clinic Florida, Jacksonville, FL, United States

**Keywords:** brain tumor surgery, direct cortical stimulation, early postoperative seizure, epilepsy, glioma, postoperative seizure, seizure

## Abstract

Early postoperative seizures (EPS) are a common complication of brain tumor surgery. EPS can lead to hemorrhage, cerebral hypoxia, increased intracranial pressure, longer hospitalization, reduced quality of life, decreased overall survival, and increased morbidity. However, there are no formal guidelines on perioperative antiseizure medication (ASM) management in patients with tumor-related epilepsy who are deemed high risk for EPS. In this study, we describe the case of a 38-year-old man with isocitrate dehydrogenase-mutant mixed glioma and two episodes of EPS manifesting with status epilepticus during prior tumor surgeries and who presented with tumor progression. The Tumor Board recommended awake craniotomy with direct electrical stimulation (DES). The patient was administered aggressive preoperative “prophylactic” ASMs by increasing the maintenance doses of lacosamide and levetiracetam by 25% 48 h before surgery. An intravenous load of fosphenytoin (20 mg/kg) was administered in the operating room before DES, followed by a maintenance dosing of 300 mg/day for 14 days. EPS did not occur, and he was discharged home on postoperative day 4. Our case illustrates that aggressive perioperative prophylactic ASM therapy beyond the maintenance ASM regimen can be considered in patients with tumor-related epilepsy at risk of EPS.

## Introduction

Early postoperative seizures (EPS), often categorized as acute symptomatic seizures (occurring within 7 days of an inciting event) (1), are a common complication of brain tumor surgery (2–4). EPS can lead to intracranial hemorrhage, cerebral hypoxia, and increased intracranial pressure and can result in longer hospitalization, reduced quality of life, decreased overall survival, and increased morbidity (5, 6). Therefore, understanding the cases of those at higher risk of EPS is crucial, because an intervention with antiseizure medication (ASM) treatment to prevent EPS could have potential implications on outcomes (6). While the rate of incidence of EPS in seizure-naive brain tumor patients undergoing craniotomy and resection has been reported to range from 3% to 18% (7), the risk of EPS is higher in those with a history of preoperative seizures (PRS) (8). The other risk factors for EPS following craniotomy for tumor resection are poorly described but may include prior EPS, a recent increase in seizure burden, non-gross total resection, perioperative brain injury, younger age, persistent postoperative neurological deficits, and ASM polytherapy (2, 4, 6, 9).

Several studies have assessed the benefit of ASM prophylaxis in patients undergoing craniotomy for tumor resection to prevent EPS, focusing primarily on seizure-naive patients (7, 10–13). However, these studies did not assess those undergoing awake craniotomy with direct electrical stimulation (DES) for cortical mapping. Overall, the evidence has not consistently supported the indiscriminate use of ASM prophylaxis in patients undergoing brain tumor surgery to prevent EPS (7, 10). This includes data from randomized controlled studies (10). The design of previous studies, as well as the need to consider the complicated predisposing and pathophysiological factors in tumor-related epilepsy, could be limitations in previous studies, and therefore, future studies should focus on patients with higher risk (7, 10). Therefore, some patients could benefit from treatment (11). Significantly, there are no guidelines regarding the use of perioperative ASM prophylaxis beyond baseline ASM therapy in preventing EPS among high-risk patients with tumor-related epilepsy. Herein, we present an illustrative case with a history of recurrent EPS with status epilepticus (SE), treated with aggressive *prophylactic* perioperative ASM therapy, which prevented another episode of early postoperative SE (pSE) following awake craniotomy with DES.

## Case description

**Figure 1:**
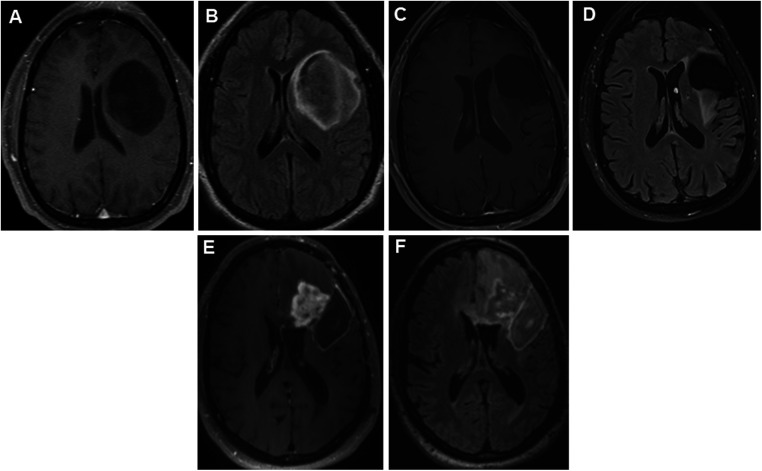
Panels of serial preoperative axial postcontrast T1 and FLAIR images in a 35-year-old patient with recurrent left hemispheric glioma. Axial postcontrast (**A**) and FLAIR (**B**) images showing a non-enhancing large left frontal tumor prior to first resection in 2012. Nine years later, he presented with expansile T2 hyperintense (**C**) tissue with minimal punctate enhancement (**D**) along the superior margin of the resection cavity within the left middle frontal gyrus. Interval tumor progression 2 years later, with progressive confluent nodular heterogeneous, solid mass-like enhancement (**E**) with surrounding unenchanting T2 hyperintensity (**F**) in the left frontal lobe at the anterior and superior margins of the previous left frontal opercular region resection cavity. FLAIR, fluid-attenuated inversion recovery.

**Figure 2:**
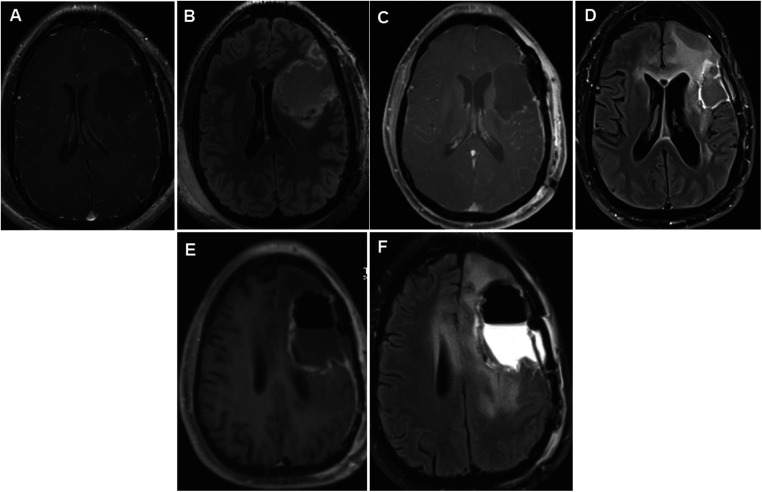
Panels of serial postoperative axial postcontrast T1 and FLAIR images in a 35-year-old patient who underwent repeated glioma resections for recurrent left hemispheric glioma. Axial postcontrast (**A**) and FLAIR (**B**) images showing fluid collection in the left frontal lobe surgical cavity on postoperative day 0 following the first glioma resection in 2012. Following the second surgery, early postoperative axial images taken on postoperative day 0 showed vascular and gyral enhancement (**C**) and decreased and increased FLAIR signal intensity (**D**) in the surgical cavity. Following the third surgery 2 years later, on postoperative day 0, axial images showed a region of unresectable enhancement in the left periventricular white matter and corpus callosum (**E**) and a hyperintense T2 FLAIR signal surrounding the resection cavity extending across the corpus callosum into the right frontal lobe (**F**). FLAIR, fluid-attenuated inversion recovery.

A 35-year-old right-handed man with a history of left frontocentral isocitrate dehydrogenase 1 (IDH1) mutant WHO grade 3 glioma, status-post gross total resection, and adjuvant chemoradiation therapy was referred to our center for the evaluation of glioma-related drug-resistant epilepsy. He had been diagnosed 7 years prior with glioma-related epilepsy and treated with levetiracetam (Figures 1A,B). He had also undergone a gross total resection with awake craniotomy with DES at another facility. Details of intraoperative electrocorticography (iECoG) and DES were unavailable, but a brain MRI on postoperative day 0 showed gross total resection (Figures 2A,B). The postoperative course was complicated by pSE, which led to a prolonged intensive care unit (ICU) course. Post-discharge, he became seizure-free on levetiracetam monotherapy until 3 years later when focal seizures recurred. Brain MRI was repeated at an undisclosed regular interval during this period, which showed no evidence of tumor progression despite recurrent seizures, prompting referral to our center.

A repeat brain MRI at our center showed subtle tumor progression, and our tumor board was convened, recommending a second brain tumor surgery. Prior to surgery, brain MRI was repeated (Figures 1C,D). He underwent awake craniotomy with DES. During surgery, neuro anesthesia was utilized based on our institution's standard protocol during awake craniotomy (14, 15). Briefly, this entailed premedication with 2 mg midazolam and 50 μg of fentanyl. Bilateral scalp blocks were given administering injections of 0.5% ropivacaine in the supraorbital, auriculotemporal, and occipital nerves bilaterally. Brain mapping was performed using an Ojemann cortical stimulator (Integra LifeSciences, Princeton, NJ, USA), with stimulation applied for 1–3 s at a pulse duration of 0.5 ms and a biphasic wave pulse rate of 50 Hz up to a maximum of 6 mA intensity or until eloquent functions were identified, and sometimes until afterdischarges were elicited. During his second surgery, iECoG demonstrated no epileptiform activity, and electrical stimulation was not performed. On postoperative day 0, another brain MRI was performed, which demonstrated gross total resection (Figures 2C,D). On postoperative day 1, the patient started having repetitive focal aware motor seizures that progressed to pSE despite intravenous lorazepam, levetiracetam, lacosamide, and valproic acid. He was then transferred to the ICU, intubated, and sedated using ketamine infusion under continuous electroencephalogram (EEG) monitoring. His pSE resolved, and he was transitioned to clonazepam and oral ketamine. Subsequently, he was extubated and maintained on oral ketamine, clonazepam, levetiracetam, and lacosamide. He was discharged on postoperative day 23 to an inpatient rehabilitation facility. Following discharge, ketamine and clonazepam were slowly weaned. He also received an adjuvant course of chemotherapy.

Breakthrough seizures occurred 14 months later. A repeat brain MRI showed tumor progression, and chemotherapy was restarted. Seven months later, another brain MRI revealed further tumor progression. Following his tumor board re-presentation, a recommendation was made for a third brain tumor surgery with awake craniotomy with DES, with another brain MRI performed shortly before surgery (Figures 1E,F). Given his history of pSE during prior surgeries, the patient received aggressive preoperative “prophylactic” ASM. His oral lacosamide and levetiracetam maintenance doses were increased by 25% 48 h before surgery. Moreover, an intravenous load of fosphenytoin (20 mg/kg) was administered in the operating room prior to DES, followed by a maintenance dose of oral phenytoin 300 mg/day for 14 days. During his third surgery, iECoG demonstrated no epileptiform activity, and electrical stimulation was not performed. A brain MRI on postoperative day 0 demonstrated a region of enhancement in the left periventricular white matter and corpus callosum, with a T2 hyperintense signal around the resection cavity that extended to the right frontal lobe through the corpus callosum (Figures 2E,F). His early postoperative course was uneventful without EPS. He was discharged home on postoperative day 4 with plans to wean phenytoin over the following 2 weeks. At the 1-month follow-up, seizures did not recur, and he remained on a maintenance ASM regimen of levetiracetam and lacosamide. A brain MRI with perfusion was performed, which showed persistent tumor progression. Table 1 summarizes both the preoperative and postoperative course of our patient following his repeated brain tumor surgeries. Moreover, Figures 1 and 2 provide serial pre- and postoperative brain MRIs that were obtained prior to and following the three surgeries for recurrent left hemispheric glioma in our patient.

**Table 1:** Summary of the timeline in a patient with a history of two episodes of early postoperative seizure during repeated brain tumor surgery for recurrent glioma.

** **	**6/2013**	**5/2020**	**6/2022**
Preoperative seizure burden	Infrequent breakthrough seizure	Occasional breakthrough seizure	Occasional breakthrough seizure
Perioperative ASM regimen	Maintenance ASM continued	Maintenance ASM continued	Levetiracetam and lacosamide dosing increased by 25% for 48 h prior to surgery; fosphenytoin loading intraoperatively; phenytoin 300 mg/day for 2 weeks
Direct cortical stimulation	Yes	Yes	Yes
Extent of resection	Gross total	Gross total	Gross total
Postoperative ASM regimen	Levetiracetam	Levetiracetam, lacosamide, clonazepam, ketamine	Levetiracetam, lacosamide
EPS with status epilepticus	Yes	Yes	No
Length of hospital stay (ICU)	7 days (3 days)	23 days (7 days)	4 days (1 day)
Duration of an inpatient rehabilitation facility	14 days	28 days	None
Pathology	Anaplastic mixed glioma, IDH-1 wild type, no deletion of 1p/19q	IDH1 mutant WHO grade 3 astrocytoma	IDH1 mutant WHO grade 4 astrocytoma no CDKN2A/B deletion; MGMT-not methylated

ASM, antiseizure medication; CDKN2A/B, cyclin-dependent kinase inhibitor 2A/B; EPS, early postoperative seizure; ICU, intensive care unit; IDH1, Isocitrate dehydrogenase 1; MGMT, O6-methylguanine-DNA methyltransferase; WHO, world health organization.

## Discussion

We report the case of a patient with a history of two prior episodes of pSE in whom another episode of pSE was successfully prevented with aggressive preoperative “prophylactic” ASM therapy during his third brain tumor surgery. Our case illustrates that adjusting maintenance ASMs and a brief course of perioperative ASM prophylaxis can potentially prevent EPS, including pSE, thereby significantly lowering morbidity, mortality, and duration of hospital stays associated with EPS among high-risk patients undergoing repeat brain tumor surgeries with DES.

Postoperative seizures are considered EPS when they occur within 7 days of brain tumor surgery and delayed postoperative seizures (DPS) when they occur at >7 days (15, 16). While EPS are thought to be acute symptomatic seizures attributed directly to resection and craniotomy and not necessarily to the glioma itself, DPS is comparable to unprovoked seizures related to an underlying epileptogenic tendency, which may or may not be related to the glioma (16). Moreover, EPS have different triggers and risk factors from PRS and DPS (12, 17). As in our case, a recent preoperative history of a high burden of PRS (3) is a risk factor for EPS that needs to be considered in perioperative ASM management. This is particularly crucial given the increasing use of awake craniotomy with DES due to its established benefit in tumor surgery (18) and the possibility that both cortical manipulation during surgery and DES may be additive to increase the risk of EPS (2). Perioperative complications, including brain hemorrhage, may also increase the risk of EPS (6), although in the case of our patient, there was no evidence of complications related to surgery, as demonstrated by postoperative MRI studies (Figure 2).

Multiple studies and systematic reviews have assessed the benefit of ASM prophylaxis in patients undergoing craniotomy for tumor resection to prevent EPS. However, these studies predominantly reported data in seizure-naive patients at low risk (7, 10, 11). One of the systematic reviews found that ASM prophylaxis provided a clinical benefit that was statistically significant in reducing EPS (11). However, others did not support the routine use of prophylactic ASMs perioperatively in the setting of craniotomy in those without a history of PRS. One study of ASM dosing adjustments during awake craniotomy for tumor resection in patients with a history of PRS demonstrated the benefit of monotherapy or dual therapy ASM “prophylaxis” around the time of surgery (5). Another study reported reduced intraoperative seizures with an intravenous load of ASMs at surgery in patients with and without a history of epilepsy (19). However, information regarding perioperative ASM prophylaxis in preventing EPS related to awake craniotomy with DES in high-risk patients is lacking.

In our patient, we opted to use an adjunct intraoperative loading of fosphenytoin and a brief 14-day course of postoperative phenytoin and to optimize the maintenance ASM regimen. Phenytoin was chosen because it offered the advantage of being loaded intravenously, and fosphenytoin because of its demonstrated efficacy in acute symptomatic seizures in craniotomy for brain tumor surgery (20). Further, valproate may increase the risk of perioperative bleeding in brain surgeries, although this is controversial (21). Furthermore, our patient received levetiracetam and valproic acid during his prior brain tumor surgeries and despite this, he developed EPS. Although levetiracetam is the preferred drug for postoperative seizure prophylaxis in seizure-naive patients undergoing brain tumor surgery, a brief use of adjunct ASMs with sodium channel blocking properties, including fosphenytoin as well as lacosamide (which the patient was already receiving), could potentially prevent EPS in high-risk patients (22). The increasing use of awake craniotomy and DES in tumor surgery makes understanding ASM prophylaxis in these cases important. Future prospective studies are needed to identify the optimal timing, duration, and type(s) of ASM to prevent EPS in high-risk patients undergoing brain tumor surgery.

Patients with a history of high-grade glioma-associated PRS and EPS may be at high risk of developing EPS, including pSE. For these patients, it would be imperative to consider preoperative maintenance ASM dose optimization and a brief use of adjunct intraoperative intravenous loading of ASM with a brief postoperative treatment course, to reduce the risk of EPS and subsequent morbidity, including prolonged hospitalizations and persistent postoperative neurological deficits (6) that may delay the initiation of further tumor treatment, including chemotherapy. Following awake craniotomies, EPS may also be emotionally troubling for patients and their families because they raise concerns about a failed operation or surgical complications. Further, preventing EPS and limiting the duration of hospitalization and the need for inpatient rehabilitation are essential in patients with higher-grade gliomas, as they can lead to significant reductions in the quality of life, given their already shortened expected life span (5). Moreover, with shrinking healthcare funding and increased scrutiny of postoperative morbidity, prevention of EPS is crucial.

While acknowledging the limitations of a single case report, we encourage other physicians to consider a similar approach as illustrated herein for patients at high risk of EPS undergoing repeat brain tumor surgeries. Prevention of EPS can shorten the length of stay and influence the quality of care during hospitalization. Future prospective studies are needed to evaluate prophylactic preoperative ASM dosing optimization and perioperative ASM loading in high-risk patients undergoing craniotomy to prevent EPS and its associated morbidity.

## Data Availability

The raw data supporting the conclusions of this article will be made available by the authors, without undue reservation.
